# Letter to the Editor: Re-exploration of the optimal threshold for restrictive transfusion after hip fracture: a multicenter prospective cohort

**DOI:** 10.1097/JS9.0000000000002749

**Published:** 2025-08-25

**Authors:** Hui Shang, Xianqiang Liu, Guocai Li

**Affiliations:** a Department of Anesthesiology, Shenzhen Hospital (Fu Tian) of Guangzhou University of Chinese Medicine, Shenzhen, People’s Republic of China; b Graduate School, Medical School of Chinese PLA, Beijing, People’s Republic of China

*Dear Editor,*

I have read with great interest the multicenter prospective cohort study published in your journal regarding restrictive transfusion thresholds in patients with hip fractures^[1]^. The authors confirmed the safety of lowering the red blood cell transfusion threshold to 7 g/dL in patients without cardiovascular disease (CVD), but indicated that applying this threshold in patients with concomitant CVD may increase the risk of mortality. This study undoubtedly provides valuable data for clinical transfusion strategies; however, I believe there are several issues worthy of further discussion and reflection. I hereby present the following critical comments for peer consideration and discussion.

**1. Study design and inherent bias**

Although this study is a prospective cohort study, it is not a randomized controlled trial (RCT), making selection bias unavoidable. The authors mention that blood supply shortages might be random events but do not specify how potential confounding factors, especially disease severity, comorbidities, and treatment differences, were controlled. These factors could significantly influence transfusion decisions and prognosis.

**2. Sample size and statistical power insufficiency**

Subgroup analyses of patients with CVD and chronic kidney disease (CKD) showed a trend toward increased mortality in the transfusion threshold <7 g/dL group; however, some results did not reach statistical significance. Additionally, the CKD subgroup had insufficient statistical power (power = 0.592), which limits the generalizability of the conclusions. Future studies are recommended to increase sample sizes or to specifically design RCTs targeting high-risk subgroups to obtain more robust evidence.

**3. Rationality of patient grouping and threshold settings**

The study sets transfusion thresholds at 7 g/dL and 8 g/dL and groups patients based on baseline CVD status. However, adjustments for dynamic changes in anemia severity and other clinical parameters (e.g., cardiac function scores, bleeding volume, and inflammatory markers) were lacking. CVD patients are heterogeneous, and grouping solely by baseline CVD status may mask important risk factors. Furthermore, the lower threshold group had a significantly reduced transfusion rate; it remains to be explored whether potential organ hypoxia caused by insufficient transfusion was adequately monitored or managed.

**4. Follow-up duration and lack of long-term outcomes**

The authors note a relatively short follow-up period, with mortality differences becoming apparent only after 6 months. Given the chronic progressive nature of cardiovascular and renal diseases, short-term follow-up may be insufficient to comprehensively assess the impact of transfusion strategies on long-term organ function and quality of life. Longer-term follow-up is recommended in future research to systematically evaluate chronic complications and patient functional recovery.

In summary, this study provides important references for selecting transfusion thresholds in patients with hip fractures. Although it supports restrictive transfusion in patients without CVD, it indicates potential increased risks in those with comorbid cardiovascular or renal dysfunction. Current evidence is insufficient to support widespread adoption of lower transfusion thresholds, and clinicians should make individualized assessments to carefully formulate transfusion strategies, avoiding a “one-size-fits-all” approach. I look forward to more high-quality RCTs to further clarify the optimal transfusion strategies for different patient populations. I thank the editor and authors for this study and anticipate further in-depth discussions.

## Data Availability

Not applicable.

## References

[1] Jiang D Wei M Lin S. Re-exploration of the optimal threshold for restrictive transfusion after hip fracture: a multicenter prospective cohort. Int J Surg 2025;10–97. 10.1097/JS9.0000000000002599 40434794

[2] Agha R Mathew G Rashid R. Transparency In The reporting of Artificial INtelligence – the TITAN guideline. Prem J Sci 2025;10:100082.

